# *Streptomyces tirandamycinicus* sp. nov., a Novel Marine Sponge-Derived Actinobacterium With Antibacterial Potential Against *Streptococcus agalactiae*

**DOI:** 10.3389/fmicb.2019.00482

**Published:** 2019-03-13

**Authors:** Xiaolong Huang, Fandong Kong, Shuangqing Zhou, Dongyi Huang, Jiping Zheng, Weiming Zhu

**Affiliations:** ^1^Hainan Key Laboratory for Sustainable Utilization of Tropical Bioresources, Hainan University, Haikou, China; ^2^Key Laboratory of Marine Drugs, Ministry of Education of China, School of Medicine and Pharmacy, Ocean University of China, Qingdao, China; ^3^Open Studio for Druggability Research of Marine Natural Products, Pilot National Laboratory for Marine Science and Technology, Qingdao, China

**Keywords:** *Streptomyces tirandamycinicus*, marine sponge, antibacterial, *Streptococcus agalactiae*, *tirandamycins*

## Abstract

A novel actinobacterium, strain HNM0039^T^, was isolated from a marine sponge sample collected at the coast of Wenchang, Hainan, China and its polyphasic taxonomy was studied. The isolate had morphological and chemical characteristics consistent with the genus *Streptomyces*. Based on the 16S rRNA gene sequence analysis, strain HNM0039^T^ was closely related to *Streptomyces wuyuanensis* CGMCC 4.7042^T^ (99.38%) and *Streptomyces spongiicola* HNM0071^T^ (99.05%). The organism formed a well-delineated subclade with *S. wuyuanensis* CGMCC 4.7042^T^ and *S. spongiicola* HNM0071^T^ in the *Streptomyces* 16S rRNA gene tree. Multi-locus sequence analysis (MLSA) based on five house-keeping gene alleles (*atp*D, gyrB, rpoB, recA, trpB) further confirmed their relationship. DNA–DNA relatedness between strain HNM0039^T^ and its closest type strains, namely *S. wuyuanensis* CGMCC 4.7042^T^ and *S. spongiicola* HNM0071^T^, were 46.5 and 45.1%, respectively. The average nucleotide identity (ANI) between strain HNM0039^T^ and its two neighbor strains were 89.65 and 91.44%, respectively. The complete genome size of strain HNM0039^T^ was 7.2 Mbp, comprising 6226 predicted genes with DNA G+C content of 72.46 mol%. Thirty-one putative secondary metabolite biosynthetic gene clusters were also predicted in the genome of strain HNM0039^T^. Among them, the tirandamycin biosynthetic gene cluster has been characterized completely. The crude extract of strain HNM0039^T^ exhibited potent antibacterial activity against *Streptococcus agalactiae* in Nile tilapia. And tirandamycins A and B were further identified as the active components with MIC values of 2.52 and 2.55 μg/ml, respectively. Based on genotypic and phenotypic characteristics, it is concluded that strain HNM0039^T^ represents a novel species of the genus *Streptomyces* whose name was proposed as *Streptomyces tirandamycinicus* sp. nov. The type strain is HNM0039^T^ (= CCTCC AA 2018045^T^ = KCTC 49236^T^).

## Introduction

Marine actinomycetes, particularly marine sponge-associated actinomycetes have gained considerable attention during the last decade as a vast source of novel natural products (NPs) with variety of bioactivities, including anti-biofilm (Balasubramanian et al., 2017, 2018), anti-chlamydial (Reimer et al., 2015; Cheng et al., 2016), antimicrobial (El-Gendy and El-Bondkly, 2010; Elsayed et al., 2018), antioxidant (Grkovic et al., 2014; Cheng et al., 2016), antiparasitic (Elsayed et al., 2017), antitumor (Yi-Lei et al., 2014; Yan et al., 2017), and immunomodulatory (Tabares et al., 2011) activities. These diverse bioactive NPs are represented by alkaloids (Elsayed et al., 2017), polyketides (Schneemann et al., 2010), peptides (Cheng et al., 2017), quinolone (Cheng et al., 2016), and anthraquinones (Abdelfattah et al., 2018). Accordingly, the isolation and identification of actinomycetes from marine sponge has become into a fruitful area of research in latest years, which has subsequently led to discovering novel actinobacteria species (Abdelmohsen et al., 2014).

In recent studies, a variety of new species of actinobacteria from marine sponges were continuously identified by researchers, including *Actinokineospora spheciospongiae* (Kämpfer et al., 2015), *Marmoricola aquaticus* (de Menezes et al., 2015), *Streptomyces spongiicola* (Huang et al., 2016), *Saccharopolyspora spongiae* (Souza et al., 2017) and *Williamsia spongiae* (de Menezes et al., 2017), *Streptomyces reniochalinae* and *Streptomyces diacarni* (Li et al., 2018). Thus, marine sponges have proven to be a good habitat for novel actinomycete species (Abdelmohsen et al., 2014; Huang et al., 2016).

During our ongoing efforts to discover antibacterial agents from marine sponge-associated actinomycetes, a novel actinobacterial strain HNM0039^T^ isolated from a marine sponge sample that was collected at the coast of Wenchang, Hainan island of China, was recognized as a novel species of the genus *Streptomyces* through a polyphasic approach and its name was proposed as *Streptomyces tirandamycinicus* sp. nov. in the present study. The extract of the fermentation broth of strain HNM0039^T^ exhibited strong antibacterial activity against *Streptococcus agalactiae* (group B streptococcus) which was the causative agent for streptococcosis disease affecting various freshwater and seawater fish species worldwide, particularly Nile tilapia (Evans et al., 2015). Bioassay-guided fractionation was used to isolate bioactive compounds from the extract. In addition, the complete genome information of strain HNM0039^T^ was also analyzed to obtain further understanding on its antibacterial potential at genomic levels.

## Materials and Methods

### Isolation and Maintenance of Strain

The sponge, SP-1 (Supplementary Figure S1), was collected from the coast of Wenchang City, Hainan Province of China in May 2011. After thoroughly rinsed with sterile seawater, the sponge sample was cut into tiny pieces and homogenized in sterile seawater. The homogenate was diluted in series and spread on plates of humic acid-vitamin agar (Hayakawa and Nonomura, 1987) prepared with 50% (v/v) seawater and supplemented with K_2_Cr_2_O_7_ (100 mg/L), and cultured at 28°C for 21 days. Strain HNM0039^T^ was isolated and purified on ISP2 agar medium prepared with 50% (v/v) seawater. The purified isolate was stocked on slants of ISP2 agar at 4°C and in glycerol 20% (v/v) suspensions at −20°C.

### Phylogenetic and Genomic Analyses

Extraction of genomic DNA were carried out as described by Zhou et al. (2017). The complete 16S rRNA gene of strain HNM0039^T^ were taken from its complete genome sequence. The calculation of 16S rRNA gene sequence similarities and identification of phylogenetic neighbors were carried out using the EzTaxon-e server (Yoon et al., 2017a). The phylogenetic trees were constructed using neighbor-joining (Saitou and Nei, 1987), maximum-likelihood (Felsenstein, 1981) and maximum-parsimony (Kluge and Farris, 1969) methods with the MEGA 7.0 program (Kumar et al., 2016). Evolutionary distances for the neighbor-joining analysis were computed using Kimura’s two-parameter model (Kimura, 1980). The confidence levels of the tree topologies were estimated by bootstrap analysis on 1000 replicates (Felsenstein, 1985).

The sequences of five house-keeping genes, *atp*D (ATP synthase F1, beta subunit), *gyrB* (DNA gyrase B subunit), *rpoB* (RNA polymerase, beta subunit), *recA* (recombinase A) and *trpB* (tryptophan synthetase, beta subunit) were drawn from its complete genome sequence and the gene sequences of each locus for 16 related type strains were taken from GenBank (Supplementary Table S1). The sequences of five protein-encoding loci for each strain were concatenated by joining head-to-tail in-frame. Phylogenetic tree on the concatenated protein-coding sequences were reconstructed using the neighbor-joining (Saitou and Nei, 1987). MLSA evolutionary distances was calculated using Kimura’s two-parameter model (Kimura, 1980) from the MEGA 7.0 (Kumar et al., 2016).

DNA–DNA hybridization of strain HNM0039^T^ with its closest neighbors (*Streptomyces wuyuanensis* CGMCC 4.7042^T^ and *S. spongiicola* HNM0071^T^) was performed according to the optical renaturation methods (De Ley et al., 1970; Huss et al., 1983). Average nucleotide identity (ANI) analysis was performed using the online OrthoANI (Yoon et al., 2017b). The G+C content of strain HNM0039^T^ was calculated according to its complete genome sequence.

### Genome Sequencing and Bioinformatics Analysis of HNM0039^T^

The complete genome sequencing and assembly of strain HNM0039^T^ was performed as described previously by Zhou et al. (2018). The protein-coding gene prediction was carried out by Glimmer v3.02 (Delcher et al., 2007). Annotation of gene functions was performed by the NCBI Prokaryotic Genome Annotation Pipeline (Tatusova et al., 2016). The secondary metabolite biosynthetic gene clusters were predicted using the online antiSMASH v4.2.0 software (Weber et al., 2015).

### Chemotaxonomic Characteristics

Biomass used for chemotaxonomic analyses was obtained after growing in shake flasks of ISP2 broth at 28°C for 3–5 days. The analyses of sugars and amino acids in whole cell hydrolysates of strain HNM0039^T^ were performed following the methods of Lechevalier and Lechevalier (1980). Fatty acids of strain HNM0039^T^ were extracted and analyzed according to the procedures of Sherlock microbial identification (MIDI) system, ACTINO version 6.1 (Sasser, 1990). Menaquinones and phospholipids were analyzed according to the procedures of Minnikin et al. (1984).

### Phenotypic Characteristics

Strain HNM0039^T^ was then grown on ISP7 agar medium at 28°C for 21 days and observed using scanning electron microscopy (Hitachi; S3000). The 2-week-old cultures of strain HNM0039^T^ on standard ISP media (Shirling and Gottlieb, 1966) at 28°C were used to test its cultural characteristics. Colors of aerial and substrate mycelia, and diffusible pigments produced were determined by comparison against chips from the ISCC-NBS color charts (Kelly, 1964). The effects of pH, temperature, and NaCl on growth were observed on ISP2 medium after incubation at 28°C for 14 days. The pH range for growth was examined between 4.0 and 12.0 (in intervals of 1.0 pH unit). Temperature tolerance for growth was evaluated at 4, 15, 20, 25, 28, 37, 40, 45, and 50°C. NaCl tolerance for growth was tested in the presence of 0–10% (in intervals of 1% unit). The carbon-source utilization was determined following the methods of Shirling and Gottlieb (1966). Nitrogen source utilization was determined according to the method of Williams et al. (1983). Susceptibility to antibiotics was tested according to Huang et al. (2016). The antibiotics tested included chloramphenicol, kanamycin, gentamicin, streptomycin, nalidixic acid, penicillin G, rifampin, novobiocin, sulfamethoxazole, tetracycline, and tobramycin.

### Separation and Identification of Antimicrobial Compounds

The strain HNM0039^T^ was incubated in ISP2 broth as seed medium at 28°C for 3 days. The fermentation was carried out in 300 500-mL flasks and shaking for 7 days at 28°C and 180 rpm. Each flask contained 2.0 mL seed broth and 200 mL of fermentation medium that was prepared by adding 20 g glucose, 10 g soluble starch, 10 g peptone, 10 g yeast extract, 3 g beef extract, 2 g CaCO_3_, 0.5 g KH_2_PO_4_, 0.5 g MgSO_4_7H_2_O, 500 mL seawater and 500 mL tap water, and adjusted pH to 7.0 before sterilization. The total broth (60 L) was extracted three times with ethyl acetate (EtOAc) and the EtOAc solutions were concentrated on a rotary evaporator to dryness.

The dried EtOAc extract (30.0 g) was applied to vacuum liquid chromatography (VLC) on a silica gel column with a MeOH-CH_2_Cl_2_ (0–100%) linear gradient system to separate into six fractions (Fr. 1–6). Fr. 3 (8.0 g) with antibacterial activity was divided into five subfractions Fr. 3.1–3.5 by Sephadex LH-20 (MeOH). Then, Fr. 3.2 (860 mg) with antibacterial activity was further subjected to semipreparative HPLC (45% CH_3_CN-H_2_O) to yield pure compounds **1** (t*_R_* 12 min, 57.0 mg) and **2** (t*_R_* 7 min, 28.7 mg). The structures were identified by analyzing their NMR data.

### *In vitro* Antimicrobial Activities Assay

The anti-*S. agalactiae* assay utilized strain *S. agalactiae* HNe0 which was isolated from infected *Oreochromis niloticus* sample. *Staphylococcus aureus* GIM1.221, *Bacillus subtilis* GIM1.222, and *Escherichia coli* GIM1.223 were purchased from Guangdong Culture Collection Center. The antibacterial activities of crude extracts or purified compounds against four indicator microorganisms were evaluated in 96-well microtiter plates as previously described by Wang et al. (2018). DMSO and tobramycin were used as a negative control and positive control, respectively.

## Results and Discussion

### Phylogenetic and Genomic Analyses of Strain HNM0039^T^

**FIGURE 1:**
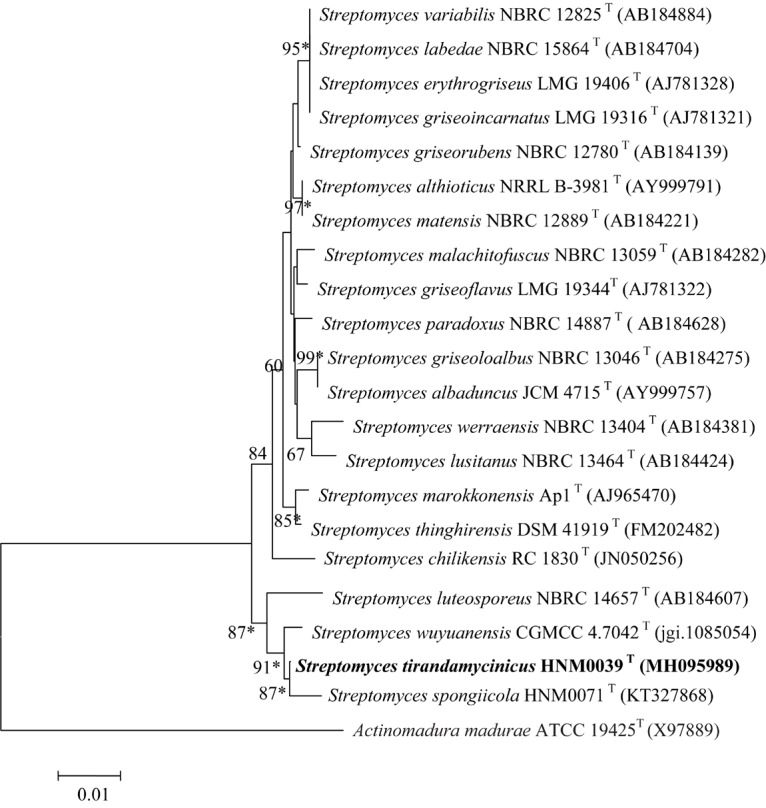
Neighbor-joining phylogenetic tree of strain HNM0039^T^ based on complete 16S rRNA gene sequences (1523 nucleotides). Asterisks indicate that the conserved branches were also recovered using maximum-parsimony and maximum-likelihood tree-making algorithms. Bootstrap values based on 1000 replications are listed as percentages; only values > 50% are given. Bar: 0.01 substitutions per nucleotide position.

The EzBioCloud analysis of complete 16S rRNA gene sequence (1523 nt) of strain HNM0039^T^ revealed that the strain was closely related to type strains assigned in the genus *Streptomyces*. Strain HNM0039^T^ shared highest 16S rRNA gene sequence similarities to *S. wuyuanensis* CGMCC 4.7042^T^ (99.38%) and *S. spongiicola* HNM0071^T^ (99.05%), and less than 99% sequence similarities to other type strains of the genus *Streptomyces*. Strain HNM0039^T^ also formed a well-delineated subclade with *S. spongiicola* HNM0071^T^ and *S. wuyuanensis* CGMCC 4.7042^T^ with a high bootstrap value (87 and 91%, respectively) in the neighbor-joining tree (Figure 1). The taxonomic status of the subclade was also supported by other tree-making algorithms.

**FIGURE 2:**
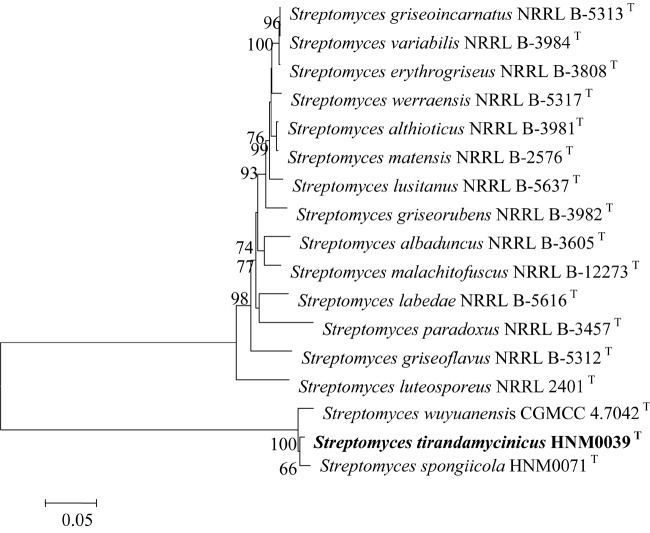
Neighbor-Joining tree based on concatenated five house-keeping genes (atpD, gyrB, rpoB, recA, and trpB) showing the position of strain HNM0039^T^ amongst its phylogenetic neighbors. Percentages at the nodes represent levels of bootstrap support from 1000 replications datasets. Only values > 50% are given. Bar: 0.05 substitutions per nucleotide position.

The close relationships between strain HNM0039^T^ with *S. spongiicola* HNM0071^T^ and *S. wuyuanensis* CGMCC 4.7042^T^ were further confirmed by the multi-locus sequence analysis (MLSA) tree (Figure 2). Strain HNM0039^T^ formed a distinct clade with *S. spongiicola* HNM0071^T^ and *S. wuyuanensis* CGMCC 4.7042^T^ with a high bootstrap value (66 and 100%, respectively) in the MLSA tree. The MLSA distance between strain HNM0039^T^ and other related type strains of *Streptomyces* species was 0.015–0.061 (Supplementary Table S2), which was well above the species level cut-off point of 0.007 proposed by Rong and Huang (2012), indicating the strain HNM0039^T^ formed a novel *Streptomyces* species.

The DNA–DNA relatedness between strain HNM0039^T^ and *S. wuyuanensis* CGMCC 4.7042^T^ and between strain HNM0039^T^ and *S. spongiicola* HNM0071^T^ were 46.5 and 45.1%, significantly less than the 70% cutoff value for recognition of genomic species differentiation (Wayne et al., 1987). Furthermore, an ANI calculated for the genomes of strain HNM0039^T^ and *S. wuyuanensis* CGMCC 4.7042^T^ (GenBank Accession No. NZ_FNHI00000000) and *S. spongiicola* HNM0071^T^ (GenBank Accession No. CP029254) was 89.65 and 91.44% respectively, which were also below the threshold value of 95∼96% for species delineation (Richter and Rosselló-Móra, 2009). Thus, these genotypic data revealed that strain HNM0039^T^ represents a novel species of the genus *Streptomyces*.

### Chemotaxonomic Analyses of Strain HNM0039^T^

The chemotaxonomic characteristics of strain HNM0039^T^ and its close phylogenetic neighbors are shown in Table 1. The predominant menaquinones of strain HNM0039^T^ were identified as MK-9(H_6_) and MK-9(H_4_). This finding is in agreement with those of closely related type strains such as *S. wuyuanensis* CGMCC 4.7042^T^ (Zhang et al., 2013) and *S. spongiicola* HNM0071^T^ (Huang et al., 2016). However, MK-9(H_8_) is not detected in strain HNM0039^T^ (Table 1), showing that strain HNM0039^T^ is different from two related type strains. The fatty-acid profile of strain HNM0039^T^ contained iso-C_16:0_ (28.69%), anteiso-C_15:0_ (18.26%), iso-C_15:0_ (17.72%), and iso-C_14:0_ (12.47%) as its major compositions. Strain HNM0039^T^ was found to consist of LL-diaminopimelic acid in cell wall, and contain glucose and galactose in whole-organism hydrolysates. Polar lipids analysis showed that the predominant phospholipids of strain HNM0039^T^ were phosphatidylglycerol, phosphatidylethanolamine, diphosphatidylglycerol, and phosphatidylinositolmannoside. Two unidentified phospholipids and one unidentified lipid were also found (Supplementary Figure S2). It was evident that the chemotaxonomic characteristics of strain HNM0039^T^ were in agreement with its assignment to the genus *Streptomyces*.

**Table 1:** Differentiation chemotaxonomic characteristics of strain HNM0039^T^, *S. wuyuanensis* CGMCC 4.7042^T^ and *S. spongiicola* HNM0071^T^.

Characteristic	1	2	3
**Major menaquinones (%)**			
MK-9 (H_4_)	41.4	8.6	23.8
MK-9 (H_6_)	58.6	59.6	65.6
MK-9 (H_8_)	–	27.0	10.6
**Major fatty acids (%)**			
Iso-C_14:0_	12.47	5.81	4.31
Iso-C_15:0_	17.72	8.79	15.35
Anteiso-C_15:0_	18.26	10.55	25.49
Iso-C_16:1_ H	–	8.30	3.72
Iso-C_16:0_	28.69	31.03	19.51
C_16:1_ ω7*c*	–	–	3.54
C_16:0_	6.71	6.33	4.11
Iso-C_17:1_ω9*c*	1.14	–	5.75
Anteiso-C_17:1_ω9*c*	–	–	3.91
Iso-C_17:0_	5.54	5.20	2.95
Anteiso-C_17:0_	4.33	–	6.86
C_17:0_ CYCLO	–	–	2.06
Major polar lipids	DPG, PG, PE, PIM	DPG, PG, PE, PI, PIM	DPG, PG, PE
Diaminopimelic acids	LL-DAP	LL-DAP	LL-DAP
Whole-cell sugars	Glu, Gal	Glu, Gal, Rib, Man	Glu, Gal, Man
DNA G+C%	72.46	71.88	72.45

*Strains: (1) HNM0039^T^; (2) S. wuyuanensis CGMCC 4.7042^T^; (3) S. spongiicola HNM0071^T^; DPG, diphosphatidylglycerol; PG, phosphatidylglycerol; PE, phosphatidylethanolamine; PI, phosphatidylinositol; PIM, phosphatidylinositol mannoside; Glu, glucose; Gal, galactose; Rib, ribose; Man, mannose. –, not detected. Data for reference strains were taken from Zhang et al. (2013) and Huang et al. (2016).*

### Phenotypic Analyses of Strain HNM0039^T^

**FIGURE 3:**
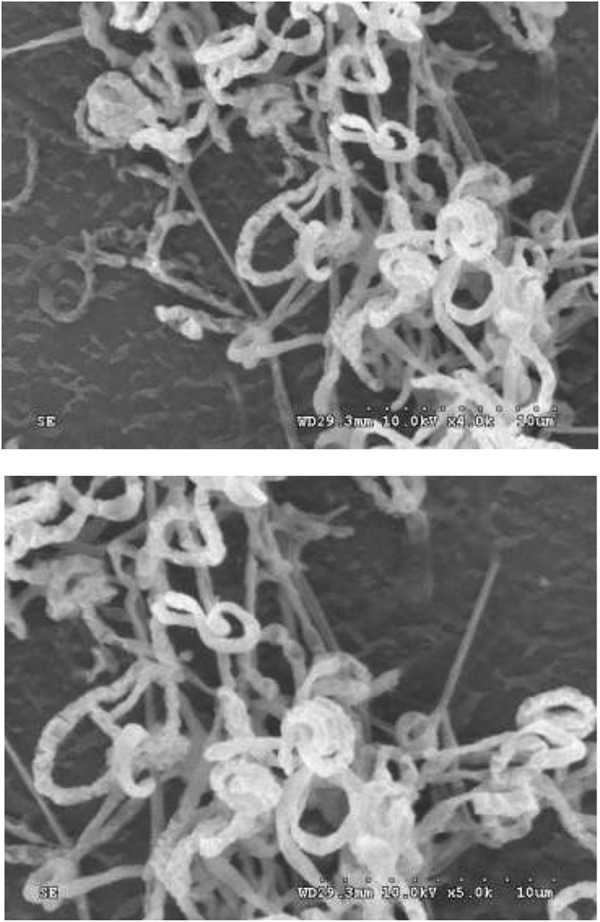
Scanning electron micrograph showing spiral spore chains of strain HNM0039^T^ after growth on ISP7 medium at 28°C for 3 weeks. Bars: 10 μm.

Strain HNM0039^T^ exhibited good growth on all of ISP media tested after 7–14 days at 28°C. The organism formed aerial hyphae on ISP4 and ISP7 agars, but none of aerial mycelia were produced on the remaining media (Supplementary Table S3). Melanoid pigments were formed on ISP6 and ISP7 agars, but diffusible pigments were not detected on the remaining media. The 21-day-old culture of strain HNM0039^T^ formed dark–brown substrate mycelia and white–gray mycelia which differentiated into curl or spiral spore chains (Figure 3). These morphological observations revealed strain HNM0039^T^ possessed the typical morphological properties of the genus *Streptomyces*. Strain HNM0039^T^ was able to survive at a pH range from 6.0 to 12.0 (optimum pH 8.0) and a temperature range from 20 to 40°C (optimum 28°C) and with 0–7% (w/v) NaCl tolerance (optimum 3%). In antibiotics sensitivity assays, the organism are resistant to chloramphenicol, gentamicin, novobiocin, nalidixic acid, penicillin G, and sulfamethoxazole, however sensitive to rifampin, kanamycin, streptomycin, tetracycline, and tobramycin. Detailed results for the physiological properties summarized in Table 2 reveal that strain HNM0039^T^ possesses several phenotypic characteristics that are clearly distinct from *S. wuyuanensis* CGMCC 4.7042^T^ and *S. spongiicola* HNM0071^T^.

**Table 2:** Differentiation physiological characteristics of strain HNM0039^T^, *S. wuyuanensis* CGMCC 4.7042^T^ and *S. spongiicola* HNM0071^T^.

Characteristics	1	2	3
**Morphology (on ISP7):**			
Aerial mycelium	White–gray	Absent	Oyster white
Substrate mycelium	Dark brown	Dark yellow	Yellowish white
Diffusible pigment	+	+	−
**Growth on sole carbon sources (1.0%,w/v)**			
L-Arabinose	−	w	−
Sucrose	+	+	+
D-Xylose	−	+	−
Myo-inositol	+	+	−
Raffinose	−	w	−
D-Galactose	+	+	+
Fructose	+	+	w
D-Glucose	+	+	+
Mannitol	−	−	−
α-L-Rhamnose	−	−	−
Ribose	+	+	+
**Growth on sole nitrogen sources (0.1%, w/v)**			
Adenine	+	+	+
L-Alanine	w	+	w
L-Arginine	+	+	+
L-Asparagine	+	+	+
Glycine	+	+	w
Hypoxanthine	−	+	−
L-Leucine	+	+	+
L-Lysine	+	+	+
L-Phenylalanine	+	+	−
L-Tryptophan	−	+	−
L-Tyrosine	w	+	−
L-Valine	+	+	+
**Growth at**			
40°C	+	+	−
pH 4	−	+	−
pH 12	+	+	−
NaCl (7%, w/v )	+	−	+

Therefore, a combination of genotypic, chemotaxonomic and phenotypic data obtained above clearly shows that the strain HNM0039^T^ should be considered as a new species within the genus *Streptomyces* and its name is proposed as *Streptomyces tirandamycinicus* sp. nov.

### Genome Properties of Strain HNM0039^T^

**FIGURE 4:**
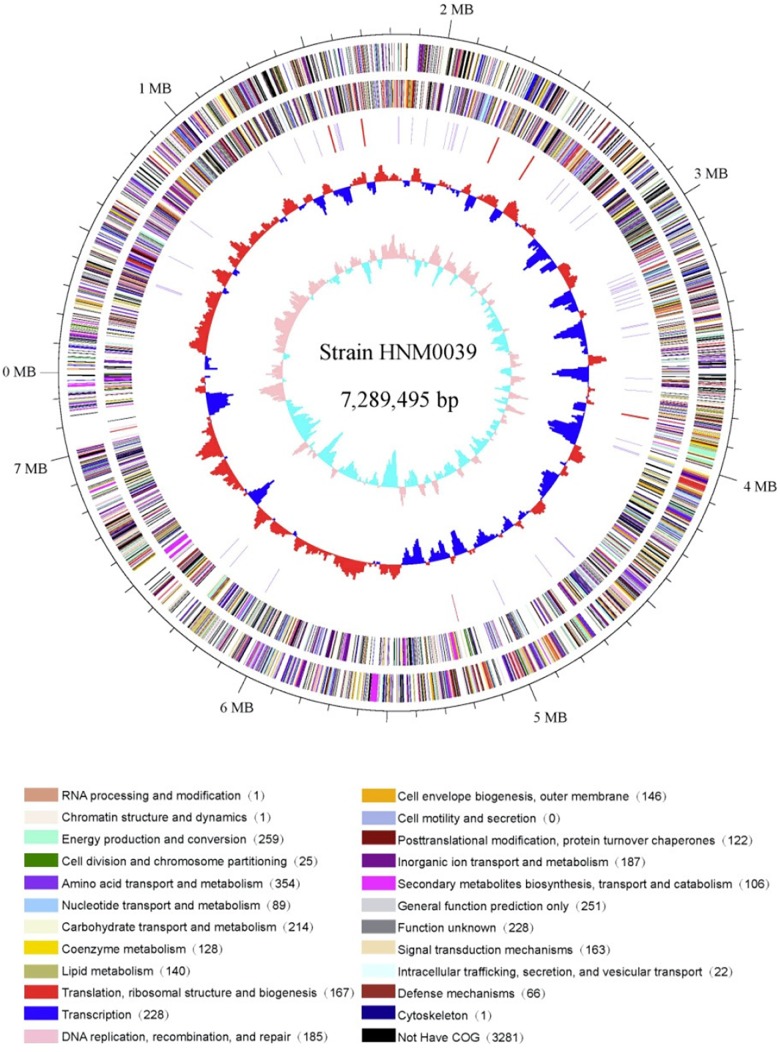
Circular genome map of strain HNM0039^T^. The genome map was made using Circos ver. 0.64 (Krzywinski et al., 2009). The outer scale is numbered in intervals of 0.1 Mbp; Circles 1 and 2 display the distribution of genes related to COG categories in the forward strand and in the backward strand respectively; Circle 3 displays the tRNA genes and rRNA genes; Circle 4 displays the GC percentage plot (red above average, blue below average); Circle 5 displays the GC skew (lime red above average, light green below average).

The complete genome of strain HNM0039^T^ consisted of a linear chromosome of 7,289,495 bp with a 72.46% G+C. Sixteen rRNA genes, 4 ncRNA genes, 68 tRNA genes, 283 pseudo genes, and 5939 protein-coding genes (CDS) were detected in the genome (Table 3). Functional analysis by clusters of orthologous genes (COGs), kyoto encyclopedia of genes and genomes (KEGG), and gene ontology (GO) revealed that 4715, 2305, and 504 out of the 5939 identified CDS were assigned to COG, KEGG, and GO categories respectively. Among the COG categories, most of predicted CDS are involved in metabolism (47.9%), followed by information storage and processing (18.9%) and cellular processes and signaling (17.7%). And the remaining (15.5%) are poorly characterized (Figure 4 and Supplementary Table S4).

**Table 3:** Genome features of strain HNM0039^T^.

Feature	Chromosome characteristics
Genome topology	Linear
Chromosome size (bp)	7,289,495
GC content (%)	72.46
Protein-coding genes	5939
Gene average length (bp)	959
Genes assigned to COG	4715
Genes assigned to KEGG	2305
Genes assigned to GO	504
rRNA genes	16
tRNA genes	68
ncRNA genes	4
Pseudo genes	283
Secondary metabolite gene clusters	31

Thirty-one biosynthetic gene clusters coding for secondary metabolites were detected in strain HNM0039^T^. These include three type 1 PKS gene clusters, one type 3 PKS gene cluster, three NRPS gene clusters, three terpene gene clusters, three bacteriocin gene clusters, two thiopeptide gene clusters, two siderophore gene clusters, one ectoine gene cluster, one linaridin gene cluster, one butyrolactone gene cluster, one melanin gene cluster, one lantipeptide gene cluster, five hybrid gene clusters, and three other gene clusters (Supplementary Figure S3).

The genome analysis further reveals that five gene clusters are involved in the production of antimicrobial metabolites, including stenothricin (Liu et al., 2014), cephamycin C (Alexander et al., 2000), streptomycin (Schatz et al., 1944), laspartomycin (Wang et al., 2011), and tirandamycin (Meyer, 1971). Amongst them, the tirandamycin gene cluster shows 100% similarity in sequence and gene order to that in *Streptomyces* sp. 307-9, including the 15 genes essential for tirandamycin biosynthesis (Carlson et al., 2010). Similarly, five gene clusters probably direct the biosynthesis of antitumor agents, including thiolutin (Huang et al., 2015), chrysomycin (Kharel et al., 2010), cosmomycin D (Rojas et al., 2014), lidamycin (Li et al., 2016), and streptazone E (Ohno et al., 2015). Other secondary metabolites are expected to be produced and excreted by strain HNM0039^T^. They include lipstatin, spore pigment, ectoine, desferrioxamine B, phosphonoglycans, carotenoid, hopene, and melanin. Finally, the products of 11 putative biosynthetic gene clusters are cryptic and unknown, indicating that strain HNM0039^T^ could be a potential candidate for novel metabolites discovery (Supplementary Figure S3).

### Antibacterial Compounds Produced by Strain HNM0039^T^

**FIGURE 5:**
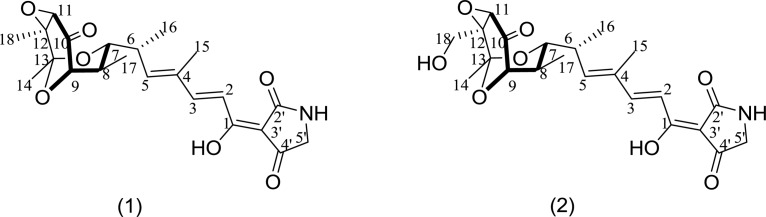
Structure of tirandamycins A **(1)** and B **(2)** from strain HNM0039^T^.

To identify the exact structures of antibacterial compounds from strain HNM0039^T^, the corresponding crude extract was fractionated by VLC on a silica gel chromatography, Sephadex LH-20 and semipreparative HPLC, which afforded two pure compounds. ESI-MS data revealed molecular ion peaks at *m/z* 456.5 [M+K]^+^ and 434.3 [M+H]^+^ for compounds **1** and **2** respectively. Their structures were confirmed as tirandamycins A (**1**) and B (**2**) (Figure 5) by comparing their NMR data (Supplementary Figures S4–S13 and Table S5) with previous published ones (Shimshock et al., 1991; Carlson et al., 2009), respectively. Compounds **1** and **2** displayed potent inhibitory activity against *S. agalactiae* HNe0 and the MIC values were 2.52 and 2.55 μg/ml (tobramycin, MIC 32 μg/mL), respectively. Compounds **1** and **2** also showed antibacterial activity against *Bacillus subtilis* GIM1.222 with MIC values of 5.5 and 6.8 μg/mL, respectively (tobramycin, MIC 0.25 μg/ml), while they were inactive against *Staphylococcus aureus* GIM1.221 and *Escherichia coli* GIM1.223 at 128 μg/mL.

Tirandamycins are a small group of actinobacterial NPs possessing a bicyclic ketal unit and a dienoyl tetramic acid moiety. In early reports, tirandamycins A and B showed antimicrobial against G^+^ bacteria and inhibited bacterial RNA polymerase (Hagenmaier et al., 1976; Reusser, 1976). Especially, Carlson et al. (2009) reported that new tirandamycin derivatives from the marine-derived *Streptomyces* species possessed activity against vancomycin-resistant *Enterococcus faecalis*. Tirandamycin B isolated from *Streptomyces* sp. 17944 efficiently killed the adult *Brugia malayi* parasites as a *B. malayi* asparagine tRNA synthetase inhibitor (Yu et al., 2011; Rateb et al., 2014). A recent study revealed that tirandamycins A and B were identified as specific inhibitors of the futalosine pathway (Ogasawara et al., 2017). The present study uncovered that tirandamycins A and B remarkably inhibited the growth of *S. agalactiae* HNe0, suggesting the potential of tirandamycins as the anti-*S. agalactiae* drug candidates.

### Description of *Streptomyces tirandamycinicus* sp. nov.

*Streptomyces tirandamycinicus* (ti.ra.nda.my.ci’ni.cus. N.L. neut. n. *tirandamycinum* tirandamycin; L. suffix *-icus-a-um* related to; N.L. masc. adj. *tirandamycinicus* related to tirandamycin, referring to the ability to produce tirandamycins).

Gram-positive, aerobic, non-motile actinobacterium forming branched substrate and aerial mycelium that differentiate into curl or spiral spore chains at mature. Grows well on all of ISP media, but melanoid pigments were formed on ISP6 and ISP7 agars. Growth occurs at pH 6–12 (optimum pH 8), at 20–40°C (optimum 28°C) and with 0–7% (w/v) NaCl tolerance (optimum 3% NaCl). D-Galactose, D-glucose, fructose, *myo*-inositol, sucrose and ribose are utilized as sole carbon sources, but D-xylose, L-arabinose, raffinose, mannitol or α-L-rhamnose are not. Adenine, glycine, L-arginine, L-alanine, L-asparagine, L-leucine, L-lysine L-phenylalanine, L-valine, and L-tyrosine are utilized as sole source of nitrogen, but not hypoxanthine or L-tryptophan. The organism are resistant to chloramphenicol, nalidixic acid, gentamicin, novobiocin, penicillin G and sulfamethoxazole, but sensitive to rifampin, kanamycin, streptomycin, tetracycline, and tobramycin.

The cell wall contains LL-diaminopimelic acid and the predominant menaquinones are MK-9 (H_4_) and MK-9 (H_6_). The major phospholipids consist of phosphatidyl-ethanolamine, diphosphatidylglycerol, phosphatidylglyc-erol, and phosphatidylinositolmannoside. The major fatty acids (>12.0%) are iso-C_16:0_, anteiso-C_15:0_, iso-C_15:0_, and iso-C_14:0_.

The type strain, HNM0039^T^ (= CCTCC AA 2018045^T^ = KCTC 49236^T^), was isolated from a marine sponge collected from the coast of Wenchang, Hainan Province of China. The 16S rRNA gene sequence of strain HNM0039^T^ has been deposited in GenBank/EMBL/DDBJ (Accession No. MH095989). The complete genome of HNM0039^T^ consists of 7,289,495 bp and the DNA G+C content of the type strain is 72.46 mol %. The complete genome sequence of HNM0039^T^ is available in GenBank under the Accession No. CP029188.

## Conclusion

The marine sponge-derived actinobacterial strain HNM0039^T^ is a novel species of the genus *Streptomyces* whose name is proposed as *Streptomyces tirandamycinicus* sp. nov. and the type strain is HNM0039^T^ (= CCTCC AA 2018045^T^ = KCTC 49236^T^). Genome analysis revealed that strain HNM0039^T^ harbored 31 gene clusters directing the biosynthesis of secondary metabolites, including tirandamycins that were identified as tirandamycins A and B active against the pathogenic *S. agalactiae* HNe0 in Nile tilapia. Therefore, strain HNM0039^T^ could be a promising candidate for treating streptococcosis disease in aquaculture.

## Author Contributions

XH and WZ conceived and designed the study. XH carried out all the experiments. FK, SZ, DH, and JZ did the data analysis. XH prepared the manuscript and WZ revised it. All authors reviewed and approved the manuscript.

## Conflict of Interest Statement

The authors declare that the research was conducted in the absence of any commercial or financial relationships that could be construed as a potential conflict of interest.
