# Supplementary material for: Streptomyces tirandamycinicus sp. nov., a Novel Marine Sponge-Derived Actinobacterium With Antibacterial Potential Against Streptococcus agalactiae
Source: Front Microbiol. 2019 Mar 13;10:482. doi: 10.3389/fmicb.2019.00482 (PMC6424883; doi:10.3389/fmicb.2019.00482)

**Supplementary Material**

### *Streptomyces tirandamycinicus* sp. nov., a novel marine sponge-derived actinobacterium [with [antibacterial](https://onlinelibrary.wiley.com/doi/abs/10.1111/jwas.12151)](https://www.frontiersin.org/articles/10.3389/fmicb.2017.00877) potential against *Streptococcus agalactiae*

***Xiaolong Huang^1,2^, Fandong Kong^2^, Shuangqing Zhou^1^, Dongyi Huang^1^, Jiping Zheng^1^ and Weiming Zhu^2,3*^***

*^1^ Hainan Key Laboratory for Sustainable Utilization of Tropical Bioresources, Hainan University, Haikou 570228, China*

*^2^ Key Laboratory of Marine Drugs, Ministry of Education of China, School of Medicine and Pharmacy, Ocean University of China, Qingdao 266003, China*

*^3^ Open Studio for Druggability Research of Marine Natural Products, Pilot National Laboratory for Marine Science and Technology (Qingdao), Qingdao, 266237, China*

** Correspondence: W. Zhu, E-mail: weimingzhu@ouc.edu.cn, Fax: +86-532-82031268; Tel: +86-532-82031268*

**Table S1.** Allele sequence accession numbers of species of the genus *Streptomyces* used for the present study

| **Species** | **Strains** | **atpD** | **gyr B** | **rec A** | **rpo B** | **trp B** |
| --- | --- | --- | --- | --- | --- | --- |
| *Streptomyces tirandamycinicus* | HNM0039 ^T^ | CP029188 | CP029188 | CP029188 | CP029188 | CP029188 |
| *Streptomyces spongiicola* | HNM0071^T^ | CP029254 | CP029254 | CP029254 | CP029254 | CP029254 |
| *Streptomyces wuyuanensis* | CGMCC 4.7042 ^T^ | NZ_FNHI01000041 | NZ_FNHI01000010 | NZ_FNHI01000001 | NZ_FNHI01000004 | NZ_FNHI01000012 |
| *Streptomyces albaduncus* | NRRL B-3605^T^ | KT384449 | KT384798 | KT385146 | KJ996741 | KT389118 |
| *Streptomyces althioticus* | NRRL_B-3981^T^ | KT384460 | KT384809 | KT385157 | KT388779 | KT389129 |
| *Streptomyces_erythrogriseus* | NRRL B-3808^T^ | KT384542 | KT384891 | KT385240 | KT388861 | KT389211 |
| *Streptomyces griseoflavus* | NRRLB-5312^T^ | KT384578 | KT384927 | KT385276 | KT388897 | KT389247 |
| *Streptomyces griseoincarnatus* | NRRL B-5313^T^ | KT384580 | KT384929 | KT385278 | KT388899 | KT389249 |
| *Streptomyces griseorubens* | NRRL B-3982 ^T^ | KT384583 | KT384932 | KT385281 | KT388903 | KT389252 |
| *Streptomyces labedae* | NRRL B-5616^T^ | KT384613 | KT384962 | KT385312 | KT388933 | KT389282 |
| *Streptomyces lusitanus* | NRRL B-5637^T^ | KJ196366 | KJ196368 | KJ196370 | KJ196372 | KJ196374 |
| *Streptomyces luteosporeus* | NRRL 2401^T^ | KT384633 | KT384982 | KT385333 | KT388953 | KT389302 |
| *Streptomyces malachitofuscus* | NRRL B-12273^T^ | KT384634 | KT384983 | KT385334 | KT388954 | KT389303 |
| *Streptomyces matensis* | NRRL B-2576 ^T^ | KT384637 | KT384986 | KT385337 | KT388957 | KT389306 |
| *Streptomyces paradoxus* | NRRL B-3457 ^T^ | KT384674 | KT385024 | KT385375 | KT388994 | KT389343 |
| *Streptomyces variabilis* | NRRL B-3984 ^T^ | KT384743 | KT385091 | KT385445 | KT389063 | KT389412 |
| *Streptomyces werraensis* | NRRL B-5317 ^T^ | KT384762 | KT385110 | KT385464 | KT389082 | KT389431 |

**Table S2.** MLSA distance values for selected strains in this study.

| Strains | MLSA distance (Kimura two-parameter) | | | | | |  |  |  |  |  |  |  |  |  |  |  |
| --- | --- | --- | --- | --- | --- | --- | --- | --- | --- | --- | --- | --- | --- | --- | --- | --- | --- |
|  | 1 | 2 | 3 | 4 | 5 | 6 | 7 | 8 | 9 | 10 | 11 | 12 | 13 | 14 | 15 | 16 | 17 |
| 1 |  |  |  |  |  |  |  |  |  |  |  |  |  |  |  |  |  |
| 2 | 0.015 |  |  |  |  |  |  |  |  |  |  |  |  |  |  |  |  |
| 3 | 0.021 | 0.027 |  |  |  |  |  |  |  |  |  |  |  |  |  |  |  |
| 4 | 0.582 | 0.59 | 0.588 |  |  |  |  |  |  |  |  |  |  |  |  |  |  |
| 5 | 0.567 | 0.573 | 0.578 | 0.056 |  |  |  |  |  |  |  |  |  |  |  |  |  |
| 6 | 0.567 | 0.573 | 0.578 | 0.056 | 0.014 |  |  |  |  |  |  |  |  |  |  |  |  |
| 7 | 0.59 | 0.595 | 0.597 | 0.071 | 0.075 | 0.069 |  |  |  |  |  |  |  |  |  |  |  |
| 8 | 0.568 | 0.574 | 0.578 | 0.056 | 0.014 | 0.002 | 0.071 |  |  |  |  |  |  |  |  |  |  |
| 9 | 0.572 | 0.579 | 0.583 | 0.063 | 0.026 | 0.034 | 0.071 | 0.034 |  |  |  |  |  |  |  |  |  |
| 10 | 0.576 | 0.58 | 0.583 | 0.075 | 0.057 | 0.05 | 0.082 | 0.05 | 0.054 |  |  |  |  |  |  |  |  |
| 11 | 0.568 | 0.574 | 0.573 | 0.049 | 0.023 | 0.024 | 0.066 | 0.024 | 0.041 | 0.063 |  |  |  |  |  |  |  |
| 12 | 0.577 | 0.584 | 0.587 | 0.101 | 0.1 | 0.099 | 0.097 | 0.097 | 0.103 | 0.105 | 0.094 |  |  |  |  |  |  |
| 13 | 0.577 | 0.582 | 0.585 | 0.042 | 0.043 | 0.045 | 0.066 | 0.045 | 0.056 | 0.069 | 0.039 | 0.096 |  |  |  |  |  |
| 14 | 0.566 | 0.572 | 0.577 | 0.055 | 0.003 | 0.016 | 0.073 | 0.016 | 0.027 | 0.059 | 0.021 | 0.098 | 0.04 |  |  |  |  |
| 15 | 0.61 | 0.611 | 0.614 | 0.079 | 0.084 | 0.08 | 0.096 | 0.081 | 0.083 | 0.081 | 0.08 | 0.121 | 0.077 | 0.084 |  |  |  |
| 16 | 0.568 | 0.574 | 0.578 | 0.056 | 0.014 | 0.002 | 0.071 | 0 | 0.034 | 0.05 | 0.024 | 0.097 | 0.045 | 0.016 | 0.081 |  |  |
| 17 | 0.568 | 0.574 | 0.573 | 0.056 | 0.018 | 0.017 | 0.071 | 0.017 | 0.037 | 0.056 | 0.022 | 0.092 | 0.044 | 0.018 | 0.082 | 0.017 |  |

Strains: 1: *Streptomyces tirandamycinicus* HNM0039 ^T^, 2: *Streptomyces spongiicola* HNM0071^T^,3:*Streptomyces wuyuanensis* CGMCC 4.7042 ^T^,4: *Streptomyces albaduncus* NRRL B-3605^T^, 5: *Streptomyces althioticus* NRRL_B-3981^T^, 6: *Streptomyces_erythrogriseus* NRRL B-3808^T^, 7: *Streptomyces griseoflavus* NRRLB-5312^T^, 8: *Streptomyces griseoincarnatus* NRRL B-5313^T^, 9: *Streptomyces griseorubens* NRRL B-3982 ^T^, 10: *Streptomyces labedae* NRRL B-5616^T^, 11: *Streptomyces lusitanus* NRRL B-5637^T^, 12: *Streptomyces luteosporeus* NRRL 2401^T^, 13: *Streptomyces malachitofuscus* NRRL B-12273^T^, 14: *Streptomyces matensis* NRRL B-2576 ^T^, 15: *Streptomyces paradoxus* NRRL B-3457 ^T^,16: *Streptomyces variabilis* NRRL B-3984 ^T^, 17: *Streptomyces werraensis* NRRL B-5317 ^T^.

**Table S3.** Cultural characteristics of strain HNM0039^T^ on various media at 28°C after 2 weeks. +, positive; -, negative.

| **Agar medium** | **Aerial mycelium** | **Substrate mycelium** | **Diffusible pigment** |
| --- | --- | --- | --- |
| ISP1 | Absent | Light yellow | - |
| ISP2 | Absent | Yellow | - |
| ISP3 | Absent | Grey pink | - |
| ISP4 | White-grey | Yellow | - |
| ISP5 | Absent | Yellow | - |
| ISP6 | Absent | Dark yellow | + |
| ISP7 | White-grey | Dark brown | + |

**Table S4.** Functional cluster of orthologous genes (COG) classification of predicted genes in strain HNM0039^T^.

| COG functional categories | CDS | of CDS |
| --- | --- | --- |
| **Information storage and processing** |  | **18.9%** |
| [A] RNA processing and modification | 1 |  |
| [B] Chromatin structure and dynamics | 1 |  |
| [J] Translation, ribosomal structure and biogenesis | 167 |  |
| [K] Transcription | 228 |  |
| [L] Replication, recombination and repair | 185 |  |
| **Cellular processes and signaling** |  | **17.7%** |
| [D] Cell cycle control, cell division, chromosome partitioning | 25 |  |
| [M] Cell wall/membrane/envelope biogenesis | 146 |  |
| [N] Cell motility | 0 |  |
| [O] Posttranslational modification, protein turnover, chaperones | 122 |  |
| [T] Signal transduction mechanisms | 163 |  |
| [U] Intracellular trafficking, secretion, and vesicular transport | 22 |  |
| [V] Defense mechanisms | 66 |  |
| [W] Extracellular structures | 0 |  |
| [Y] Nuclear structure | 0 |  |
| [Z] Cytoskeleton | 1 |  |
| **Metabolism** |  | **47.9%** |
| [C] Energy production and conversion | 259 |  |
| [E] Amino acid transport and metabolism | 354 |  |
| [F] Nucleotide transport and metabolism | 89 |  |
| [G] Carbohydrate transport and metabolism | 214 |  |
| [H] Coenzyme transport and metabolism | 128 |  |
| [I] Lipid transport and metabolism | 140 |  |
| [P] Inorganic ion transport and metabolism | 187 |  |
| [Q] Secondary metabolites biosynthesis, transport and catabolism | 106 |  |
| **Poorly characterized** |  | **15.5%** |
| [R] General function prediction only | 251 |  |
| [S] Function unknown | 228 |  |

**Table S5.** The NMR data of tirandamycin A (**1**) in DMSO-*d*_6_ and tirandamycin B (**2**) in MeOH-*d*_4_.

| position | Tirandamycin A (**1**) | | | | Tirandamycin B (**2**) | | | |
| --- | --- | --- | --- | --- | --- | --- | --- | --- |
|  | *δ*_C_ | *δ*_H_ (*J* in Hz) | COSY | HMBC | *δ*_C_ | *δ*_H_ (*J* in Hz) | COSY | HMBC |
| 1 | 173.3, C |  |  |  | 176.0, C |  |  |  |
| 2 | 116.3, C | 7.03, d (15.1) | H-3 | C-1, 4 | 117.5, CH | 7.19, d (15.0) | H-3 | C-1, 4 |
| 3 | 148.1, CH | 7.48, d (15.1) | H-2 | C-15, 3, 1 | 150.4, CH | 7.57, d (15.0) | H-2 | C-15, 3, 1 |
| 4 | 134.2, C |  |  |  | 136.2, C |  |  |  |
| 5 | 143.9, CH | 6.19, br d (10.0) | H-6 | C-15, 16, 3, 7 | 145.1, CH | 6.22, br d (9.5) | H-6 | C-15, 16, 3, 7 |
| 6 | 34.0, CH | 2.89, m | H-5, 7, 16 | C-4, 16, 5 | 35.7, CH | 2.97, m | H-5, 7, 16 | C-4, 16, 5 |
| 7 | 75.9, CH | 3.76, overlap | H-6, 8 | C-8, 6, 17 | 78.2, CH | 3.76, br d (11.5) | H-6, 8 | C-8, 6, 17 |
| 8 | 34.0, CH | 1.81, m | H-7, 9, 17 | C-9, 17, 10 | 35.7, CH | 1.97, m | H-7, 9, 17 | C-9, 17, 10 |
| 9 | 77.9, CH | 4.04, br d (6.4) | H-8 | C-9, 13, 10 | 80.0, CH | 4.04, br d (6.1) | H-8 | C-9, 13, 10 |
| 10 | 202.9, C |  |  |  | 203.4, C |  |  |  |
| 11 | 60.0, CH | 3.47, s |  | C-12, 10 | 57.9, CH | 3.62, s |  | C-12, 10 |
| 12 | 56.8, C |  |  |  | 61.0, C |  |  |  |
| 13 | 96.2, C |  |  |  | 97.2, C |  |  |  |
| 14 | 22.5, C | 1.45, s |  |  | 23.7, CH_3_ | 1.54, s |  |  |
| 15 | 12.1, CH_3_ | 1.84, s |  |  | 12.4, CH_3_ | 1.93, s |  |  |
| 16 | 16.7, CH_3_ | 1.07, d (6.9) | H-6 | C-5, 6, 7 | 17.3, CH_3_ | 1.17, d (6.5) | H-6 | C-5, 6, 7 |
| 17 | 11.1, CH_3_ | 0.63, d (6.9) | H-8 | C-7, 8, 9 | 11.7, CH_3_ | 0.74, d (6.5) | H-8 | C-7, 8, 9 |
| 18 | 15.2, CH_3_ | 1.40, s |  | C-12, 13 | 58.3, CH_2_ | 3.92, d (13.4) |  | C-12, 13 |
|  |  |  |  | C-12, 13 |  | 4.00, d (13.4) |  | C-12, 13 |
| 2' | 175.8, C |  |  |  | 176.0, C |  |  |  |
| 3' | 100.5, C |  |  |  | 102.2, C |  |  |  |
| 4' | 193.5, C |  |  |  | 196.3, C |  |  |  |
| 5' | 51.5, CH_2_ | 3.77, overlap |  | C-4' | 52.3, CH_2_ | 3.83, s |  | C-4' |

**Fig. S1**. Marine sponge SP-1, the source of *Streptomyces tirandamycinicus* sp. nov.


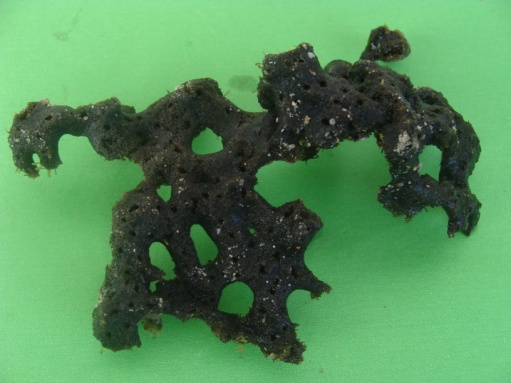


**Fig. S2.** The phospholipid profile of strain HNM0039^T^ detected with phosphomolybdic acid (a), molybdenum blue (b) and ninhydrin (c). Chloroform-methanol-water (65:25:4) was used in the first direction, followed by chloroform-acetic acid-methanol-water (80:15:12:4) in the second direction. Abbreviations: DPG, diphosphatidylglycerol; PE, phosphatidylethanolamine; PG, phosphatidylglycerol; PIM, phosphatidylinositolmannoside; PL1-2, unidentified phospholipids; L, unidentified lipid.


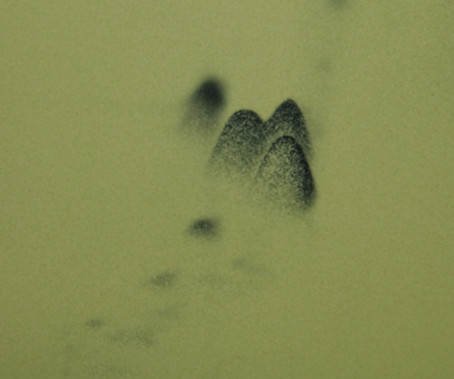

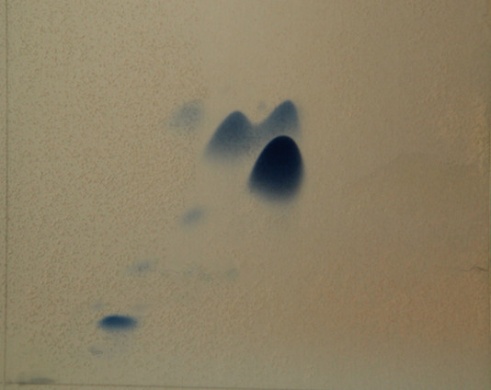

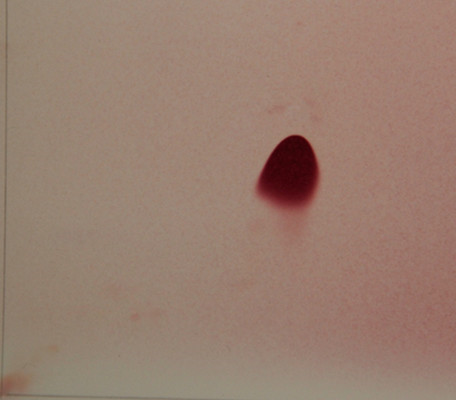


PE

DPG

DPG

PL1

**c**

**b**

**a**

2

2

2

1

1

1

PIM

PL2

PE

PIM

L

PG

PL2

PE

L

PG

PL1

**Fig. S3**. Secondary metabolite clusters are from strain HNM0039^T^ on the online antiSMASH v4.2.0

**
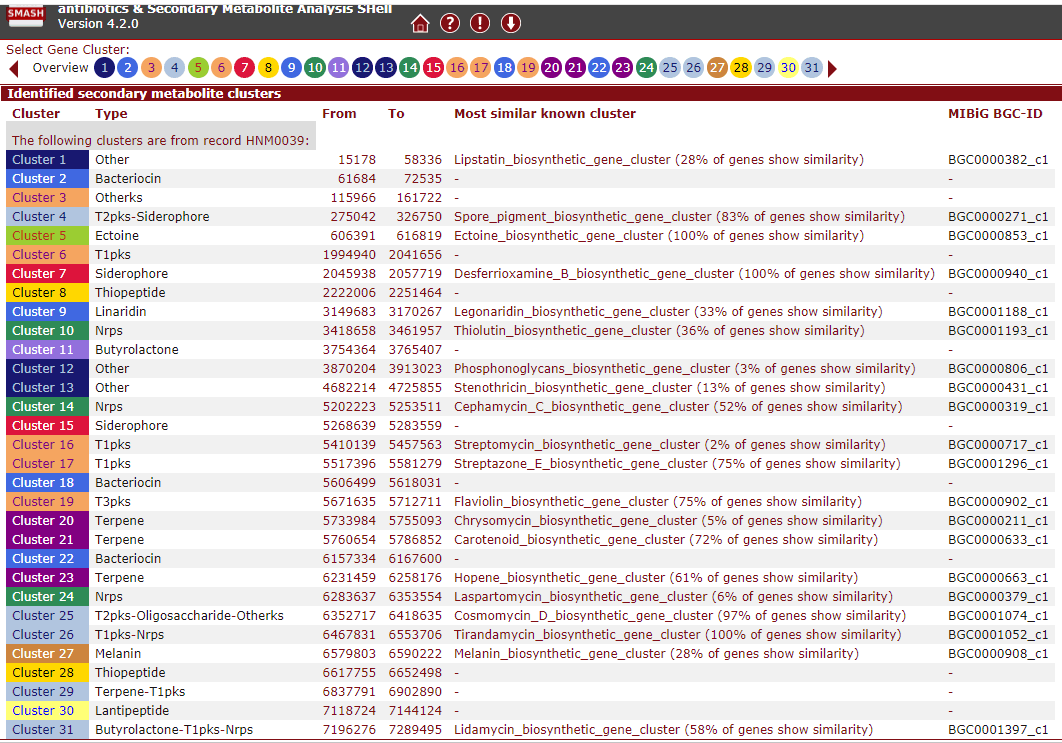
**

**Fig. S4**. The ^1^H NMR spectrum of tirandamycin A (**1**) in DMSO-*d_6_*


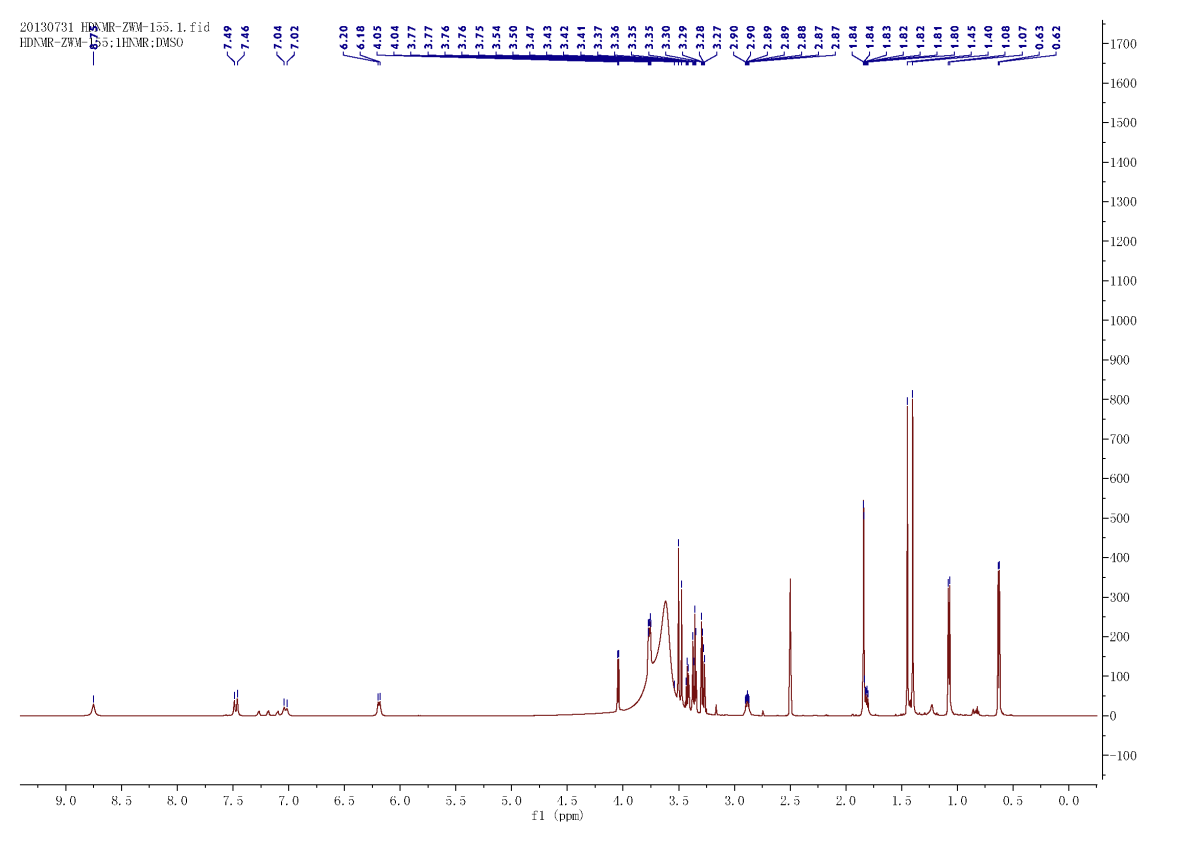


**Fig. S5**. The ^13^C NMR spectrum of tirandamycin A (**1**) in DMSO-*d_6_*


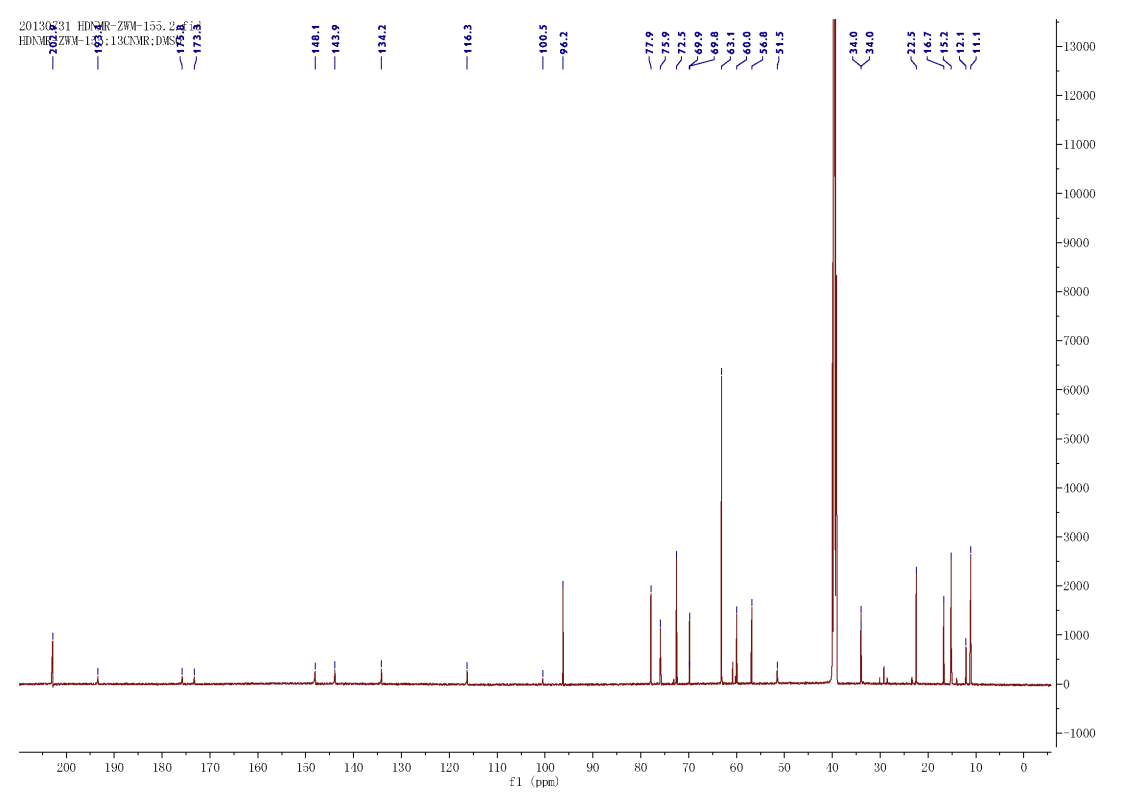


**Fig. S6**. The HMBC spectrum of of tirandamycin A (**1**) in DMSO-*d_6_*

**
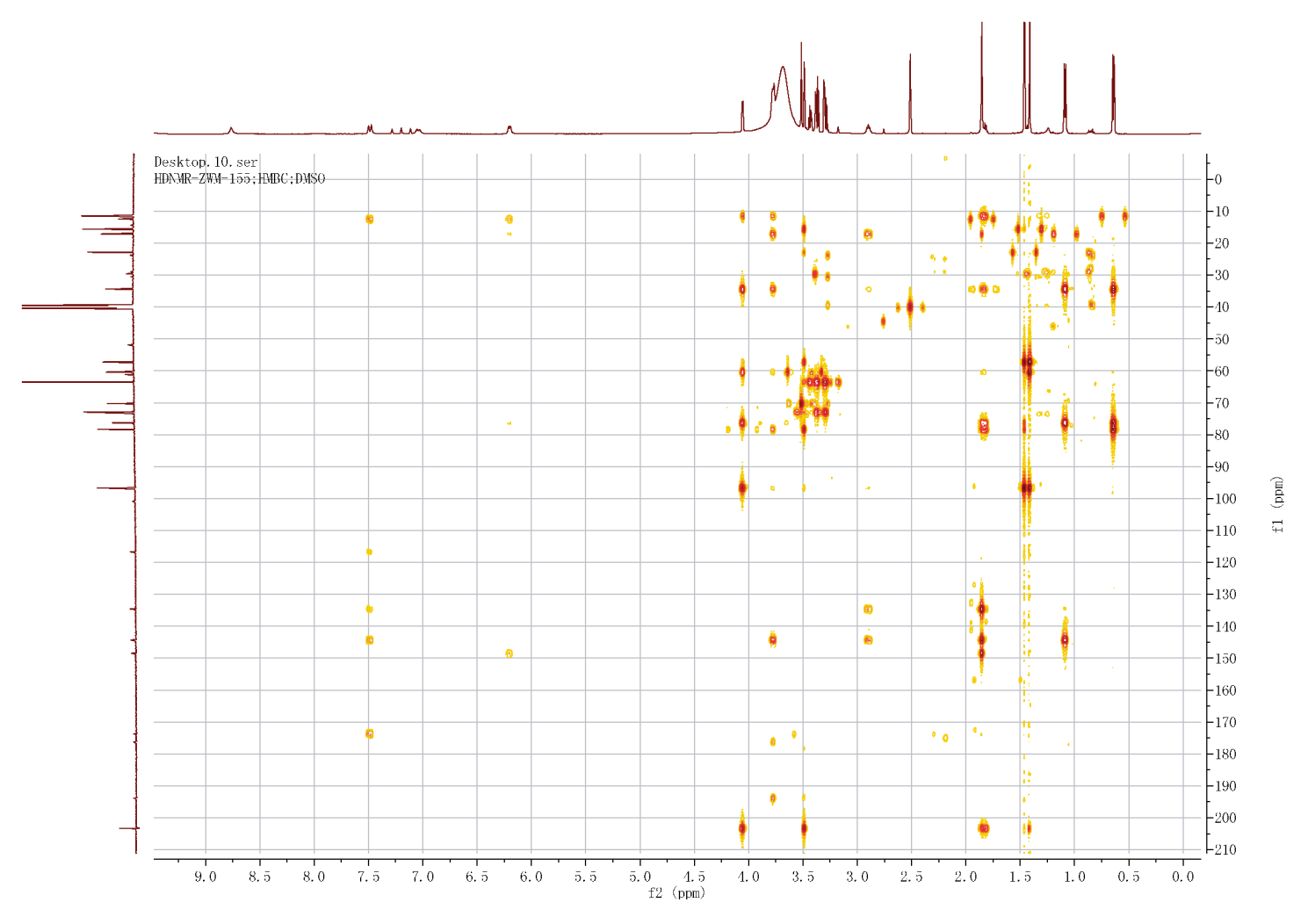
**

**Fig. S7**. The HMQC spectrum of of tirandamycin A (**1**) in DMSO-*d_6_*

**
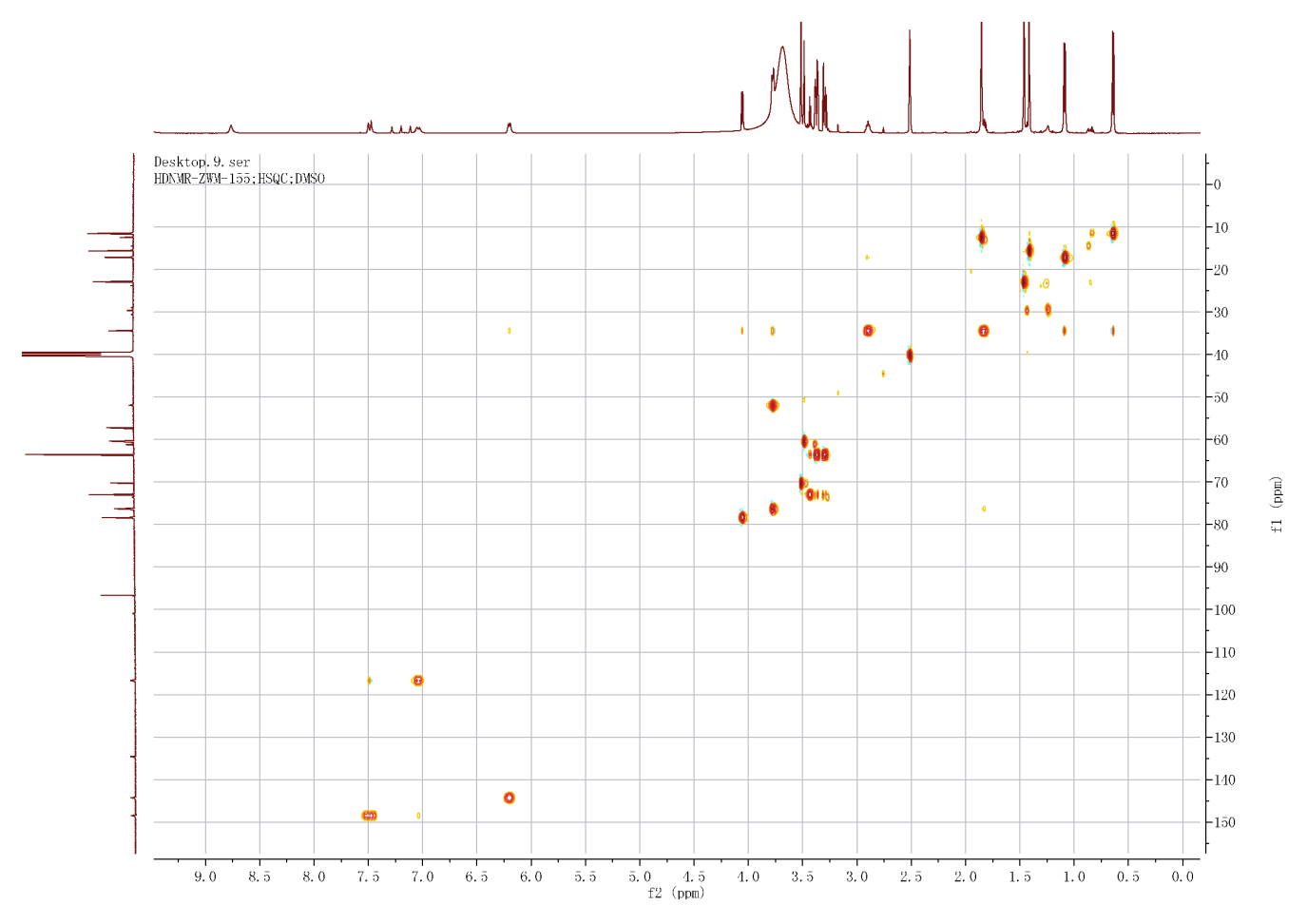
**

**Fig. S8**. The ^1^H-^1^H COSY spectrum of of tirandamycin A (**1**) in DMSO-*d_6_*

**
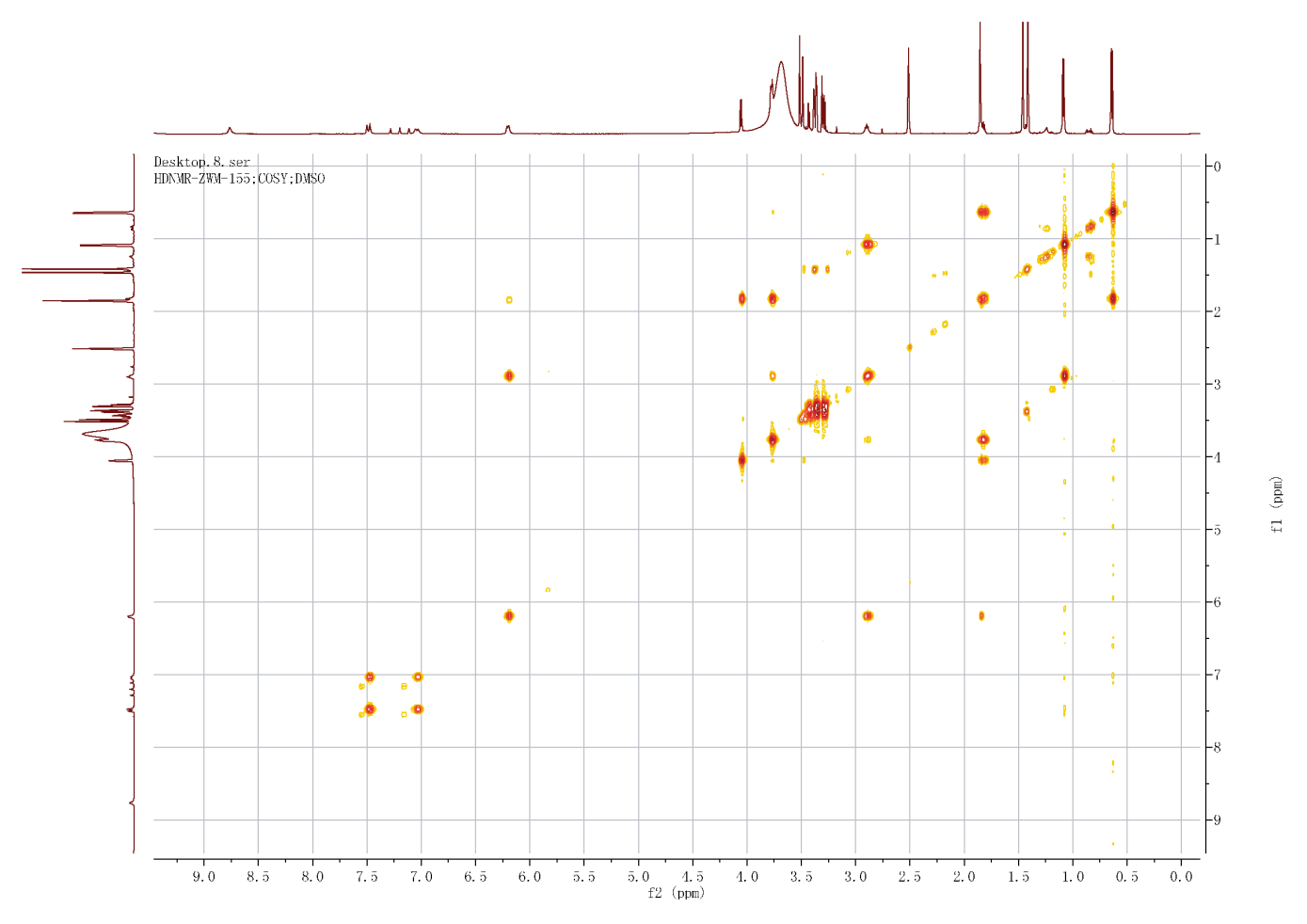
**

**Fig. S9**. The ^1^H NMR spectrum of tirandamycin B (**2**) in CD_3_OD


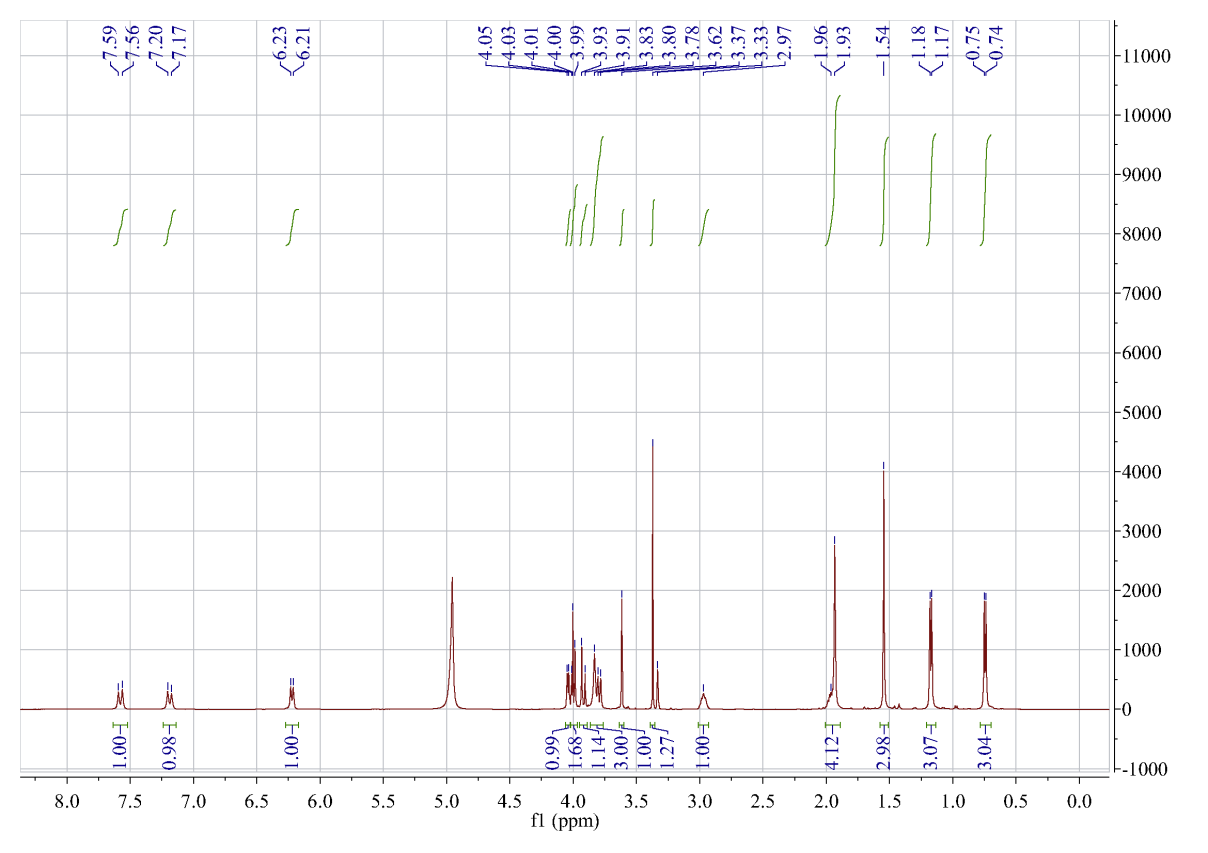


**Fig. S10**. The ^13^C NMR spectrum of tirandamycin B (**2**) in CD_3_OD


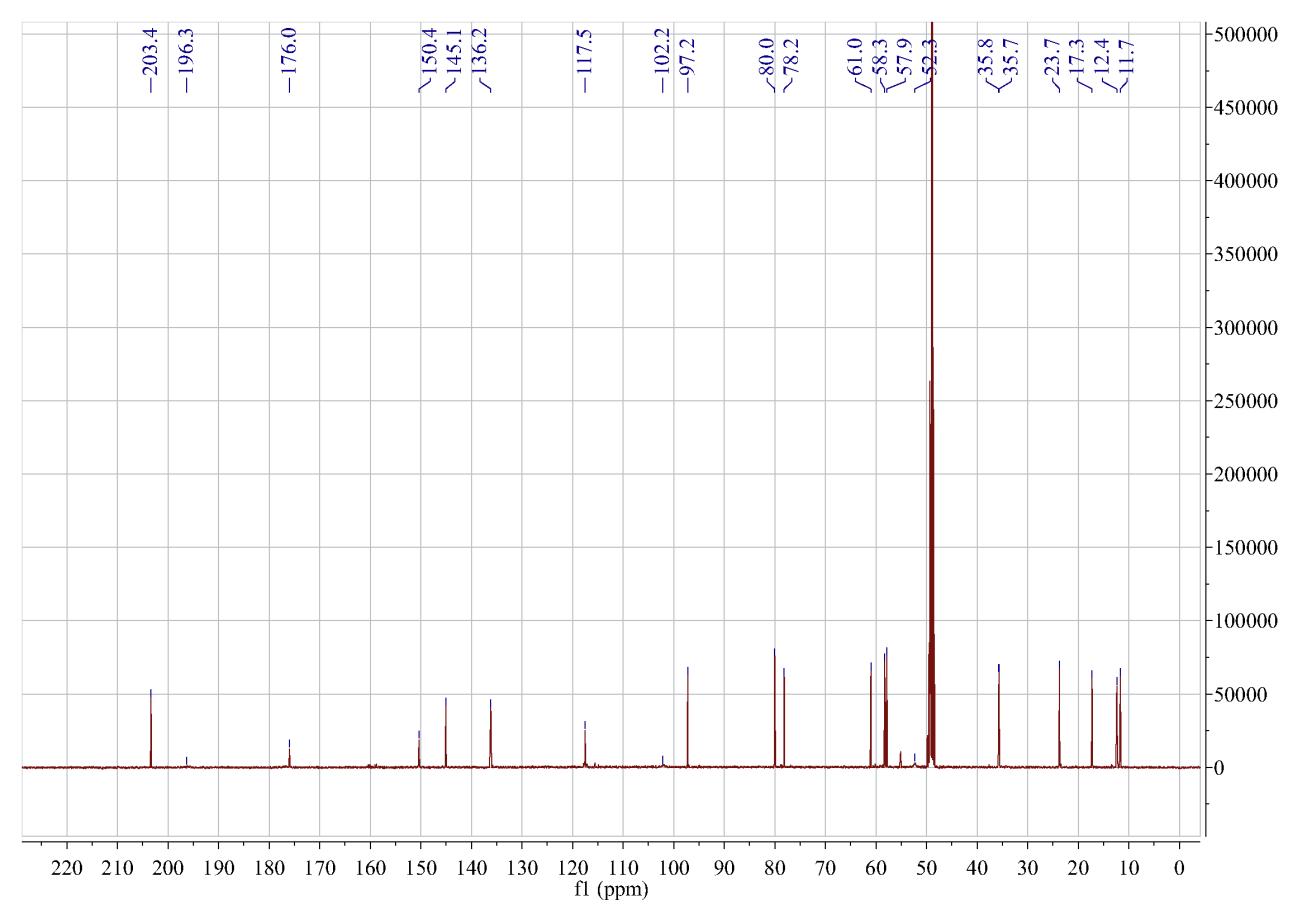


**Fig. S11**. The HMBC spectrum of of tirandamycin B (**2**) in CD_3_OD


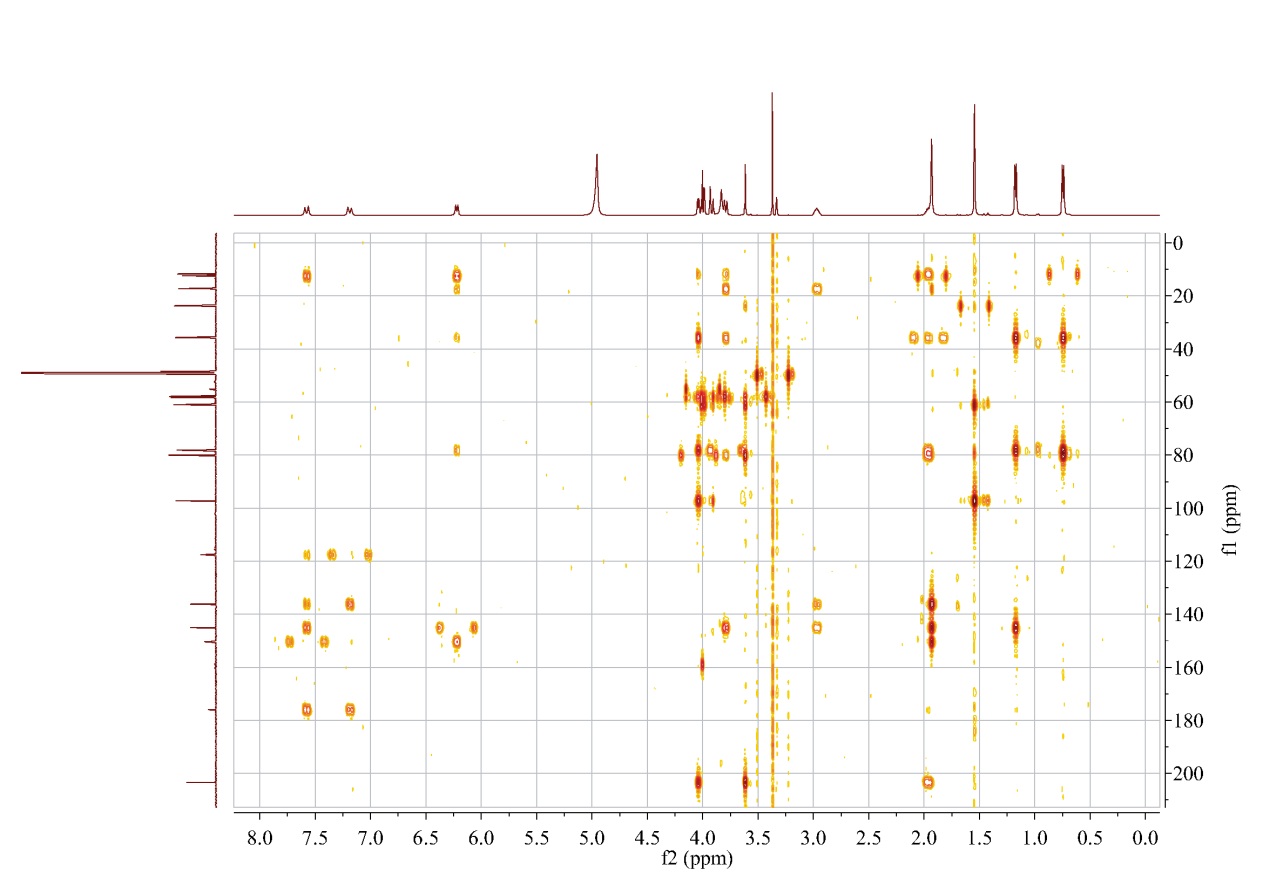


**Fig. S12**. The HMQC spectrum of of tirandamycin B (**2**) in CD_3_OD


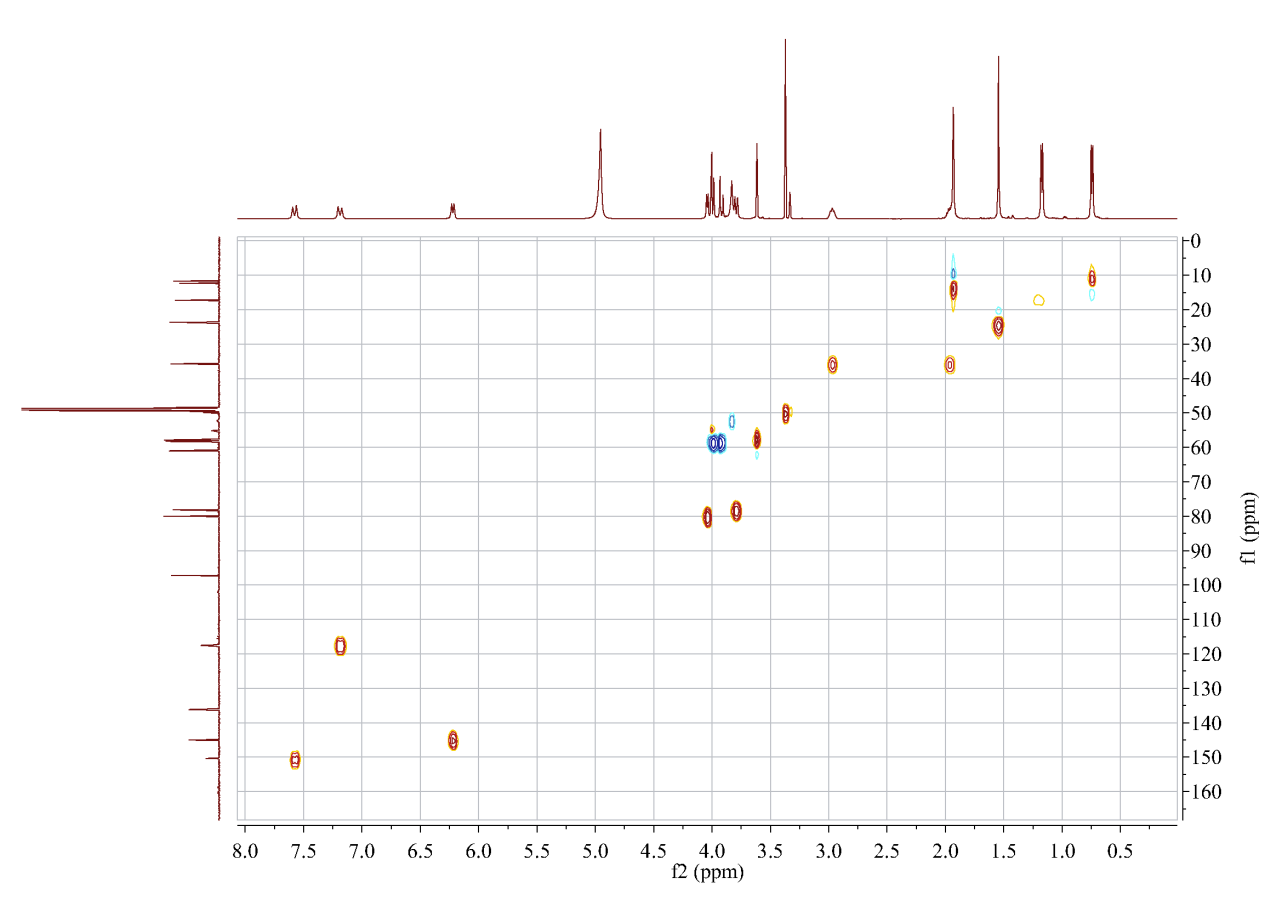


**Fig. S13**. The ^1^H-^1^H COSY spectrum of of tirandamycin B (**2**) in CD_3_OD


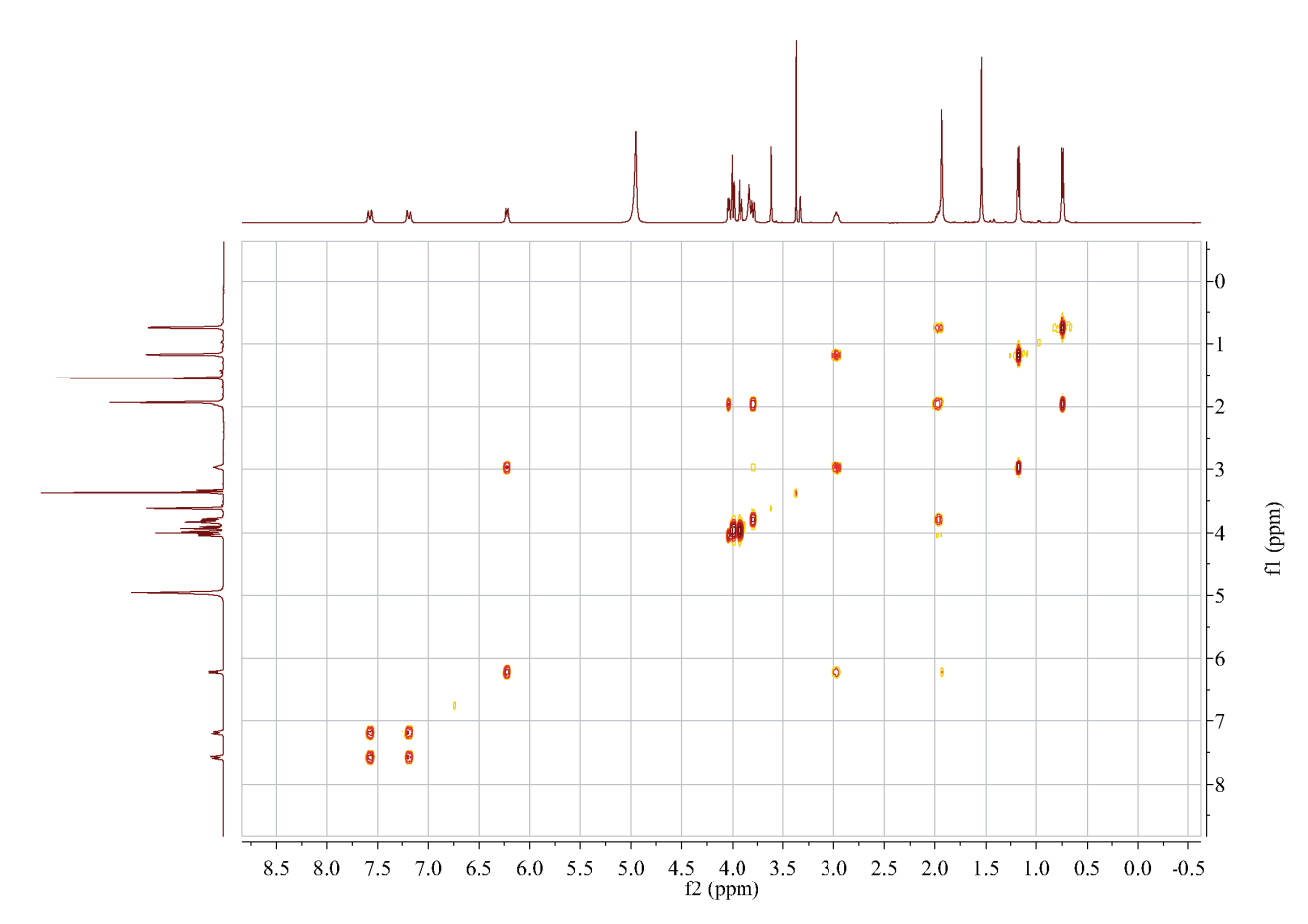

Supplement: Supplementary file 1 [file Data_Sheet_1.docx]
